# Supplementary figures and images for: Priming of myelin-specific T cells in the absence of dendritic cells results in accelerated development of Experimental Autoimmune Encephalomyelitis
Source: PLoS One. 2021 Apr 23;16(4):e0250340. doi: 10.1371/journal.pone.0250340 (PMC8064509; doi:10.1371/journal.pone.0250340)

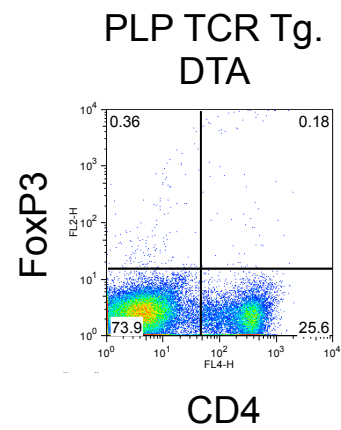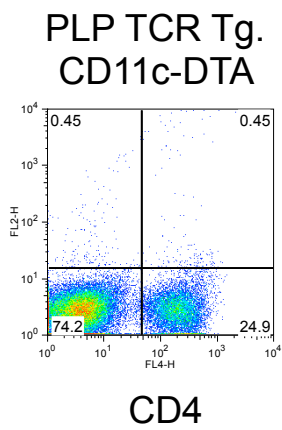

Supplement: S1 Fig — Frequencies of CD4+FoxP3+ Treg cells in PLP TCR Tg. CD11c-DTA and PLP TCR Tg. DTA control mice as determined by flow cytometric analysis of spleen cells. (PDF) [file pone.0250340.s001.pdf]

PLP TCR Tg.  
DTA

PLP TCR Tg.  
CD11c-DTA

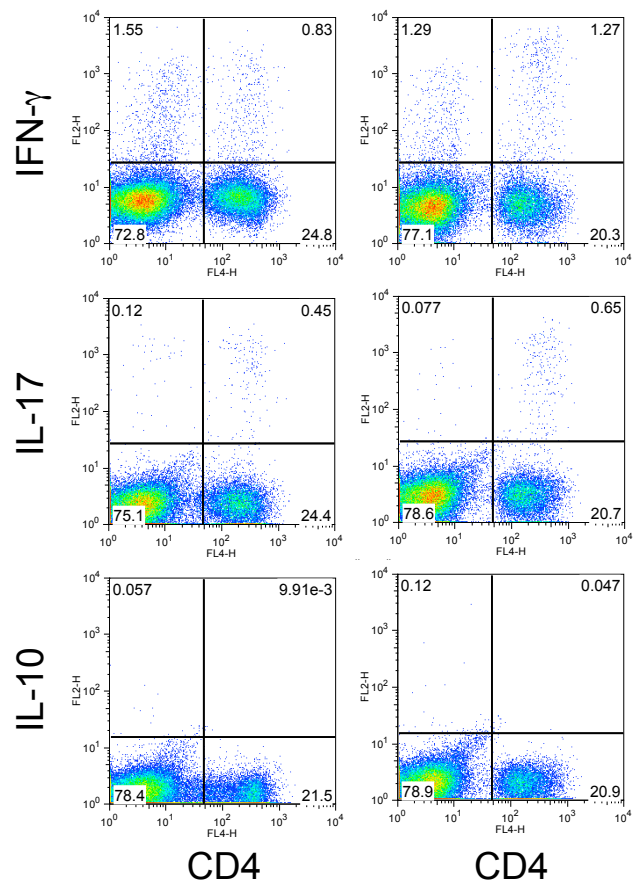

Supplement: S2 Fig — Frequencies of CD4+ and CD4- T cells, producing inflammatory cytokines (IFN-g, IL-17) or regulatory cytokine (IL-10) in response to PLP-specific stimulation in vitro from PLP TCR Tg. CD11c-DTA and PLP TCR Tg. DTA control mice, as determined by flow cytometry. (PDF) [file pone.0250340.s002.pdf]

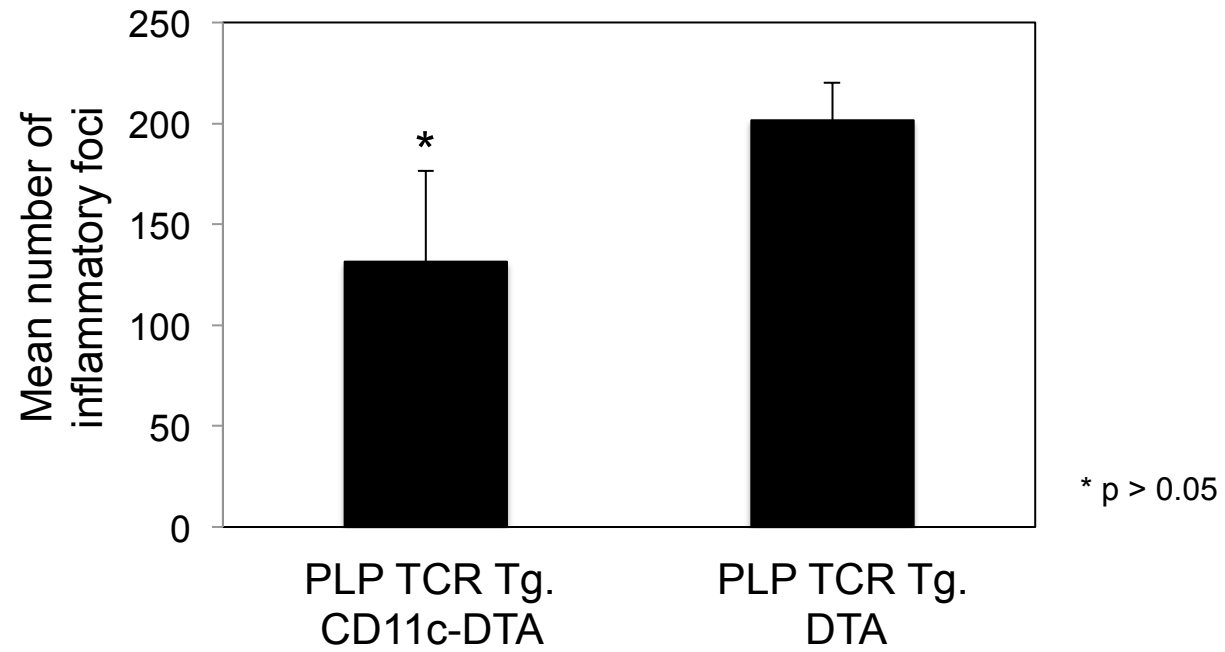

Supplement: S3 Fig — Histological severity of EAE in diseased (EAE score ≥ 3) PLP TCR Tg. CD11c-DTA and PLP TCR Tg. DTA control mice (n = 3, each) as determined by quantitation of inflammatory foci in the CNS (mean number ± SEM). * p > 0.05 (Student’s t test). (PDF) [file pone.0250340.s003.pdf]
